# Unknown Tendons, Muscles and Nerves of the Shoulder: Proposal for a Standardized Ultrasound-guided Examination, a “mini GEL” Experience

**DOI:** 10.5334/jbr-btr.917

**Published:** 2015-12-30

**Authors:** Philippe Meyer, Eric Pelé, Lionel Pesquer, Jacques Adolphe, Hervé Bard, Jean-Louis Brasseur, Christophe Courthaliac, Catherine Cyteval, Henri Guerini, Pascal Huot, Anne Miquel, Maryse Moinard, Gérald Paris, Nicolas Poussange, Alain Silvestre, Thierry Tavernier, Nicolas Wakim, Benjamin Dallaudière

**Affiliations:** 1 Centre d’Imagerie Ostéo-articulaire, Clinique du Sport de Bordeaux-Mérignac, 2 rue Négrevergne, 33700 Mérignac, FR; 2 Cabinet de radiologie, rue docteur villers, 76410 Saint-Aubin-lès-Elbeuf, FR; 3 4 Rue Léon Vaudoyer, 75007 Paris, FR; 4 Service de Radiologie, AP-HP, CHU Pitié-Salpêtrière - 91 boulevard de l’hôpital 75013 Paris, FR; 5 Imagerie Médicale Blatin, 26 r Blatin, 63000 Clermont Ferrand, FR; 6 Service de Radiologie, CHU LaPeyronie, 371 Av. du Doyen Gaston Giraud, 34295 Montpellier Cedex 5, FR; 7 Service de Radiologie B, Hôpital Cochin, AP-HP, 27 rue du Faubourg-Saint-Jacques, 75014 Paris, FR; 8 Service de radiologie, Hopital Saint Antoine, 184 r. du Fg Saint-Antoine - Paris 12e, FR; Cabinet de radiologie, 25 avenue des Sources, 69009 Lyon, FR

**Keywords:** US, shoulder, tendon, nerve, unknown

## Abstract

Thanks to its excellent spatial resolution and dynamic aspect, ultrasound of the shoulder allows an optimal evaluation of tendon, muscle and nerve’ structures in shoulder pain. Through this article and owing to inter-observer reproducibility, we will describe an ultrasound standardized protocol (posterior, anterior, global plane) in basic first ultrasounds (ie without tendon abnormality of the supra/infra spinatus, the biceps and subscapularis).

## Introduction

Nowadays the ultrasound in musculoskeletal imaging is an essential tool in patients’ diagnosis and therapeutic management. For years indeed spatial resolution of ultrasound has been increasing and to date dynamic ultrasound examination cannot be replaced by any type of imaging cross sections [1]. Though the highest majority of shoulder pains deals with «basic» tendons and muscles of the rotator cuff, secondary pains may be related to less frequent pathologies of the unknown peri musculotendinous structures.

At the posterior face of the sacpular region, the teres minor muscle, as the most distal part of the cuff is hardly isolately damaged. However it is often injured in massive rotator cuff tears and a key point in prosthetic replacement. The underlying teres major and latissimus dorsi muscles may be the site of post traumatic lesions in throwing sports. With the long head of the triceps, they can also cause pain in impingements of the Velpeau quadrilateral space, a crossing point of the axillary nerve.

At the anterior face of the shoulder, uncommon lesions of the myotendinous junction of the supraspinatus, of the short head of the biceps and coracobrachialis mainly occur on violent trauma (skydiving). The musculocutaneous nerve can also be injured at its upper portion between the two sections of the coracobrachialis muscle as the pectoralis major that can be the site of multiple pathologies affecting both the enthesis and the musculotendinous junction.

Over the cuff, the deltoid muscle is an significant abductor and shoulder stabilizer that may be injured with a large tendon rupture. It may also be a tendinopathy or myositis.

In this article, we will thus successively observe the ultrasound methods of exploration of these less known musculotendinous shoulder structures, and then nervous structures such as axillary nerve and musculo cutaneous nerve also seldom explored.

The aim of this issue is to try to standardize the ultrasound examination of the said-difficult shoulders to achieve a systematic review in case of basic first ultrasound examination.

## The Teres Minor

The teres minor inserts from the superior mid lateral scapula to the inferior posterior greater tubercle, just below the infraspinatus [2].

**Figure 1:**
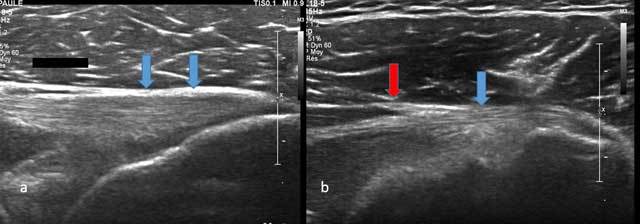
Axial view of Teres Minor with tendon (blue arrow) and musculotendinous junction (red arrow).

The tendon is examined with the arm in abduction and external rotation (similar to the infraspinatus tendon examination). Analysis is performed arms along the body in internal rotation or hand onto the contralateral shoulder (Figure 1). Sagittal cross sections are the most informative and the tendon appears to be short, hyper echoic and fibrillar.

No specific pathology of teres minor was reported though its precise exploration in posterior shoulder pain can give lots of information and helpful in case of pre prosthetic examination in extensive rotator cuff ruptures [3].

## The Teres Major

The Teres Major originates from the anterolateral plane of the scapula with an oblique up, forward and outward direction. It lies into depth of the long head of the brachial triceps and is inserted onto the medial bicipital groove through a short tendon, common to the Latissimus Dorsi [2]. At the anterior shoulder, the Teres Major is located under the subscapular as Teres Minor is located under the Infraspinatus on the posterior plane.

Exploring the Teres Major can be with arm elevation, abduction and external rotation (like a waiter with his tray) to follow continuously from its origin to its scapular humeral insertion (without changing the exploration position so as to explore the axilla). However this position is uncomfortable and difficult to maintain for the patient. Two types of injury are mainly described: tendinous avulsions and acute (baseball) or subacute (golf) injuries of the myotendinous junction in young athletes [4].

**Figure 2:**
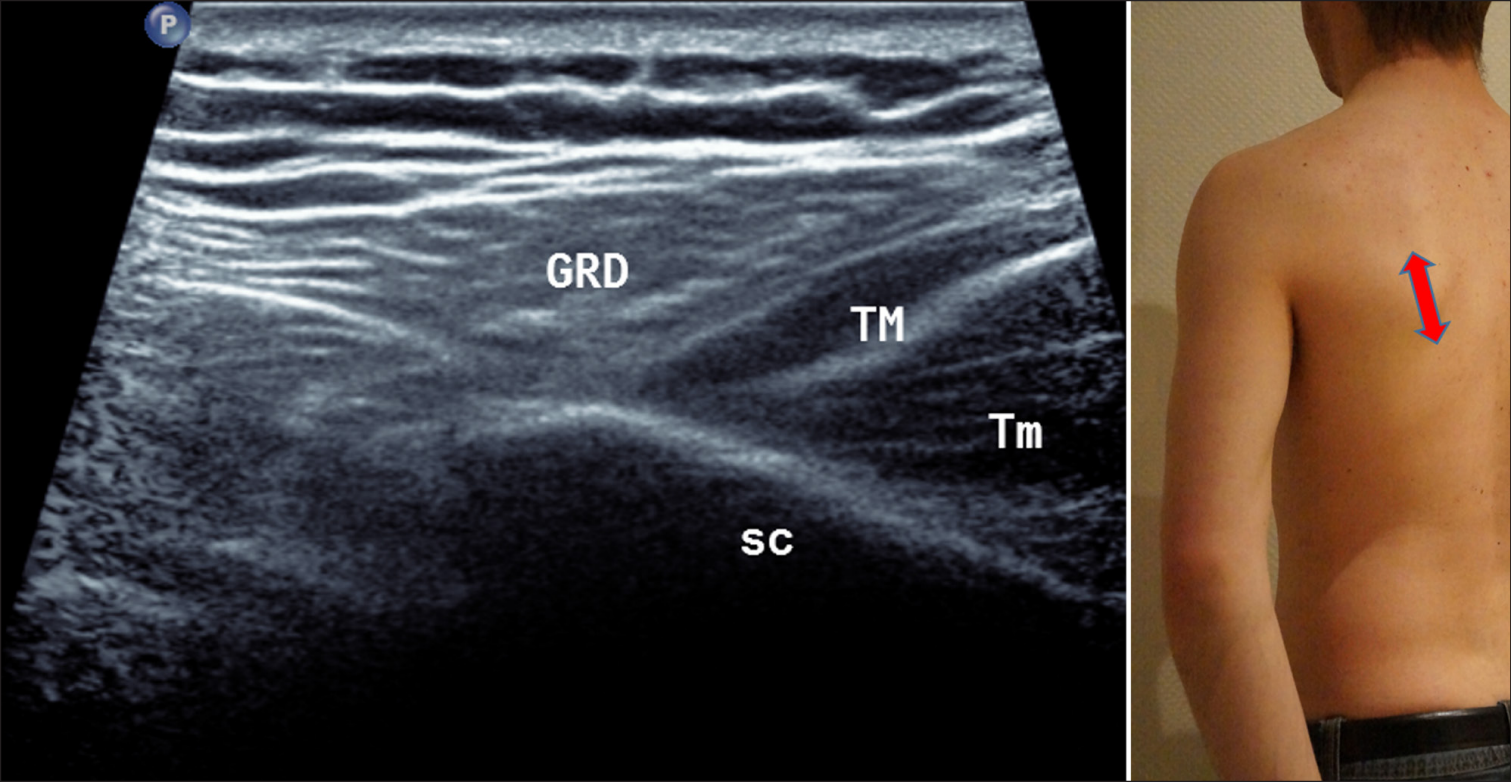
Sagittal oblique view of the scapula (Sc) in external position (red arrow). GRD: Latissimus Dorsi, TM: Teres Major, Tm: Teres minor.

**Figure 3:**
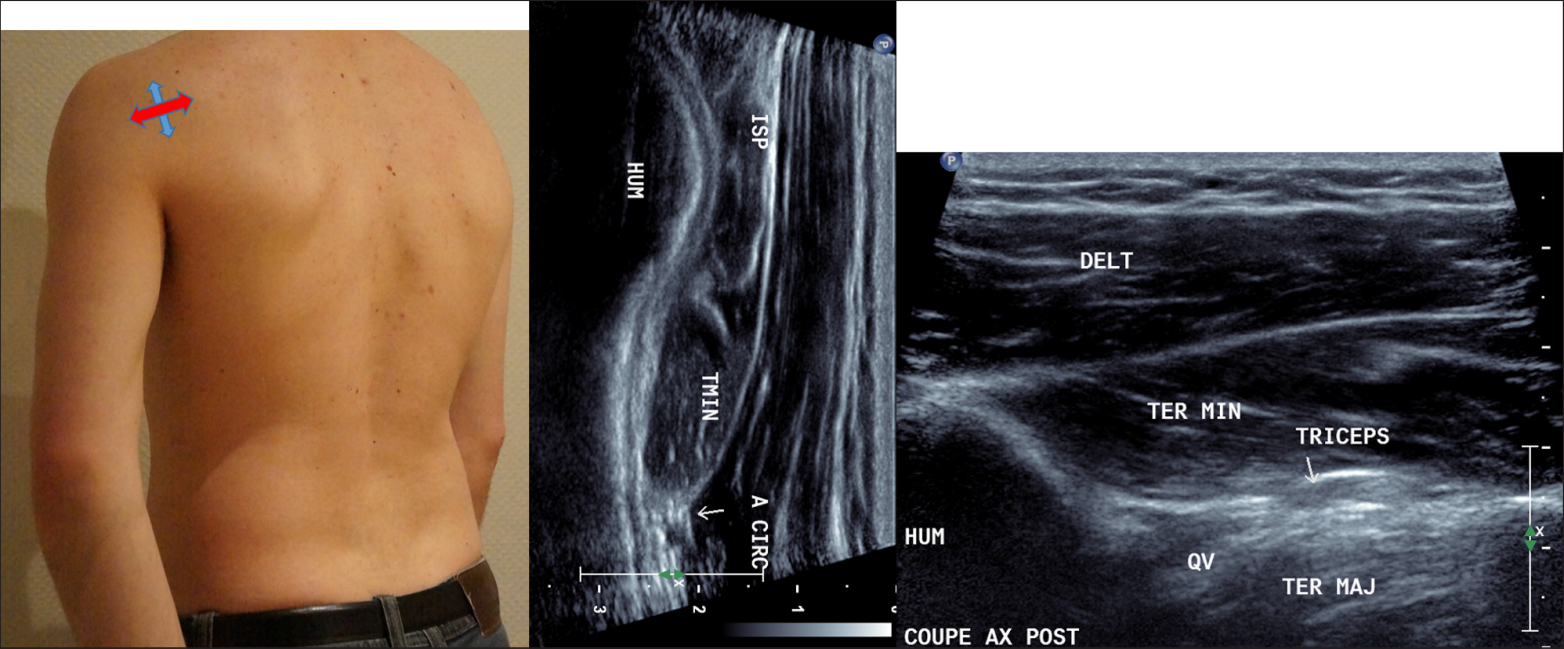
Sagittal oblique view (blue arrow) and axial intermediate view (red arrow) with HUM: Humerus, ISP: Infraspinatus, TMIN: Teres minor, A CIRC: Axillar Artery, DELT: Deltoid Muscle, TER MAJ: Teres Major, QV: Velpeau space, Triceps: Tricipal Muscle.

**Figure 4:**
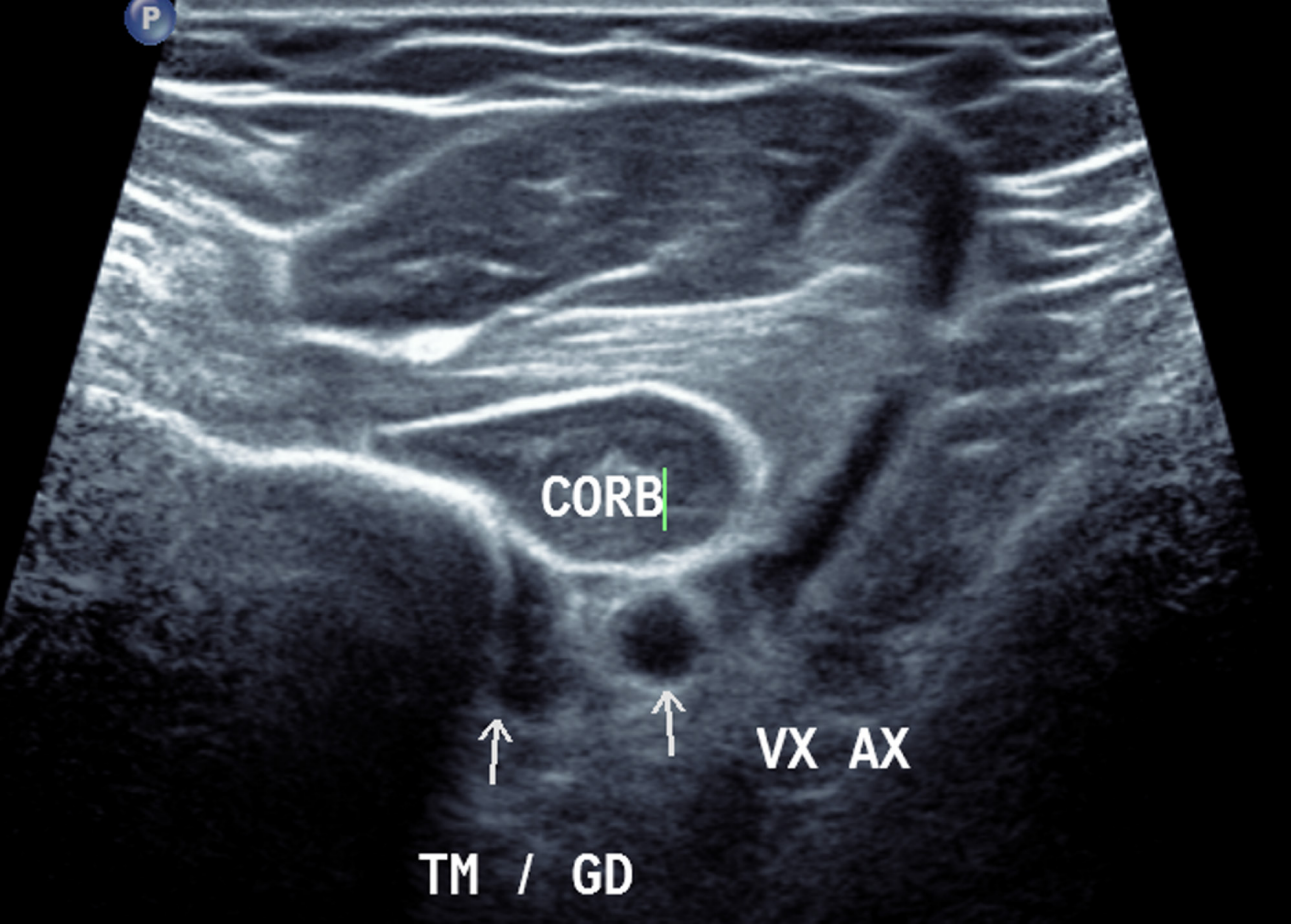
Axial distal section of the anterior and medial humerus area in external rotation. CORB: Coraco Brachal Muscle, TM/GD: Common enthesis of Teres major and Latissimus Dorsi, VX AX: Axillar Artery.

Sectional explorations seem easier to us:

- Sagittal medial cross section performed next to the lateral edge of the tip of the scapula allowing to distinguish the Teres Major from the Latissimus Dorsi (more superficial), in neutral or external rotation (Figure 2).
- Intermediate axial cross section performed in the axis of the long head of the Triceps Brachii covering the Teres Major muscle (in abduction / internal rotation similar to the infraspinatus exploration) (Figure 3).
- Axial distal section of humeral insertion in external rotation of the thick portion of the Coracobrachialis, of the brachial artery below the sub scapularis and the Pectoralis Major. The myotendinous junction of the brachial biceps only needs to be located behind the insertion of the Pectoralis Major and the probe shift to the medial arm (Figure 4).

## Latissimus Dorsi

**Figure 5:**
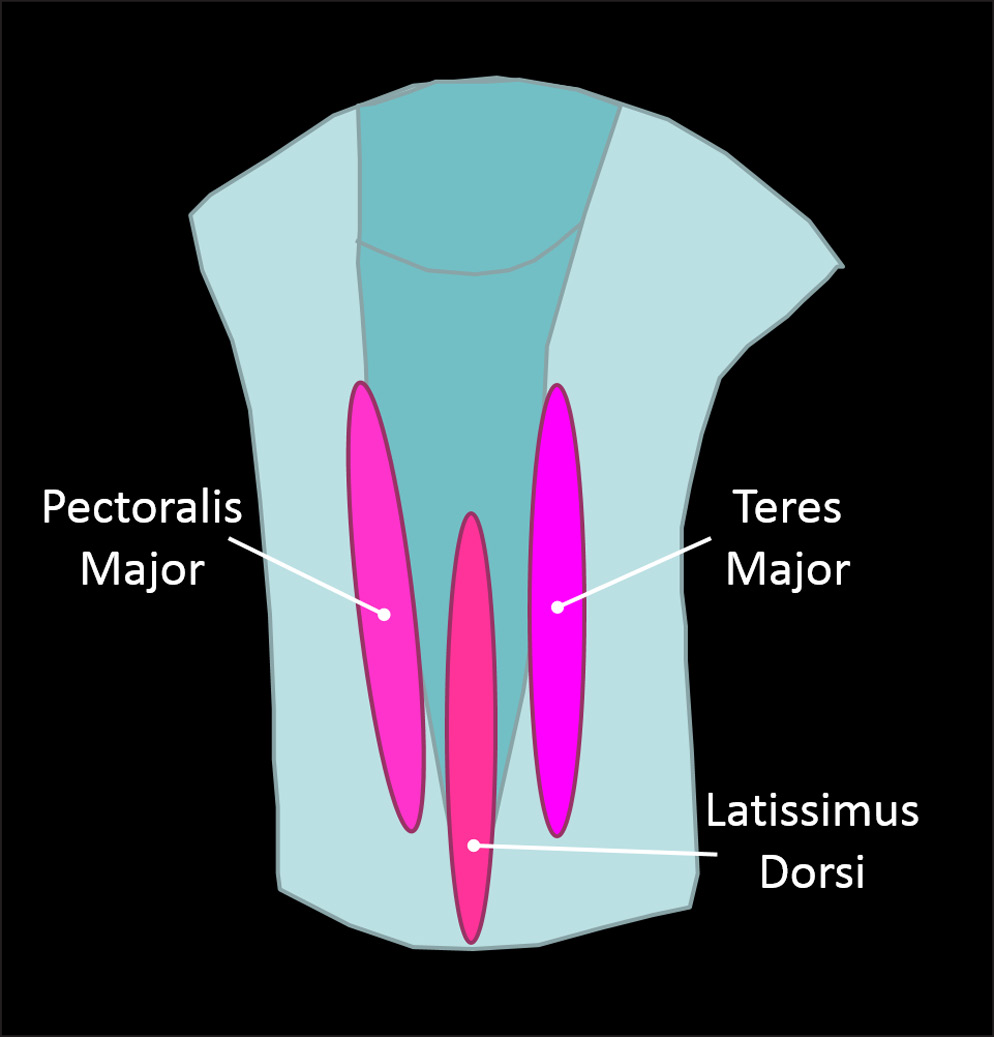
Sagittal view of the humeral diaphysis.

As adductor and medial rotator of the arm, it extends from the thoracolumbar spine (T7 – L5) and the iliac crest twisting on itself to the bottom of the intertuberosity channel between the Pectoralis Major and Teres Major muscles (Figure 5) [2].

Traumatic injuries are seldom. Concomitant injuries with the Teres Major are frequent because of their proximity, the possibility of a conjunct tendon (like hamstrings) and their equivalent function. Clinical implications are mostly minimal: the Latissimus Dorsi is used as transfer in reconstruction surgery without major clinical consequences [5].

As in Teres Major, the humeral insertion is ultrasound-guided through an anterior approach and the arm in external rotation (Figure 4). Yet the myotendinous junction is explored through an axillary posterior approach, the arm in internal rotation.

## The Quadrilateral Space Syndrome (Velpeau Quadrilateral Space)

**Figure 6:**
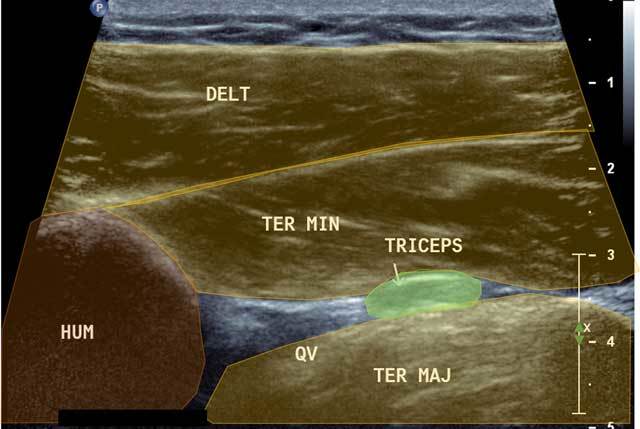
Focus in axial view of the quadrilateral space. HUM: Humerus, DELT: Deltoid muscle, QV: Quadrilateral Velpeau Space, Ter Maj: Teres Major, Ter Min: Teres Minor, Triceps: Tricipital muscle.

The Quadrilateral Space Syndrome is limited:

- Up by Teres Minor muscle
- Down by Teres Major muscle
- Inside by the long portion of the Triceps muscle
- Outside by the medial part of the proximal humeral diaphysis (Figure 6).

Both the posterior circumflex artery and the axillary nerve are held inside. The axillary nerve belongs to the posterior bundle of the Brachial Plexus and provides motor innervation of the Deltoid and Teres Minor and sensory innervation of the stump of the shoulder. It goes from the subscapular muscle and the axillary artery to the Velpeau Quadrilateral Space [6,7,8].

To analyze and locate the Velpeau Quadrilateral Space, a posterior axial cross section centered onto the Triceps Brachii tendon with the arm along the body in internal rotation (starting from an axial cross section performed in the Teres Minor axis) is needed.

**Figure 7:**
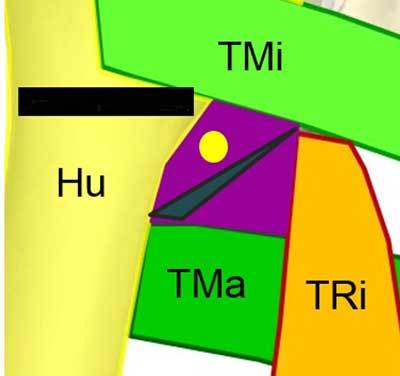
Coronal view of the QVadrilateral space. TMi: Teres Minor, TMa: Teres Major, TRi: Tricipital muscle, Yellow circle: Axillar nerve.

**Figure 8:**
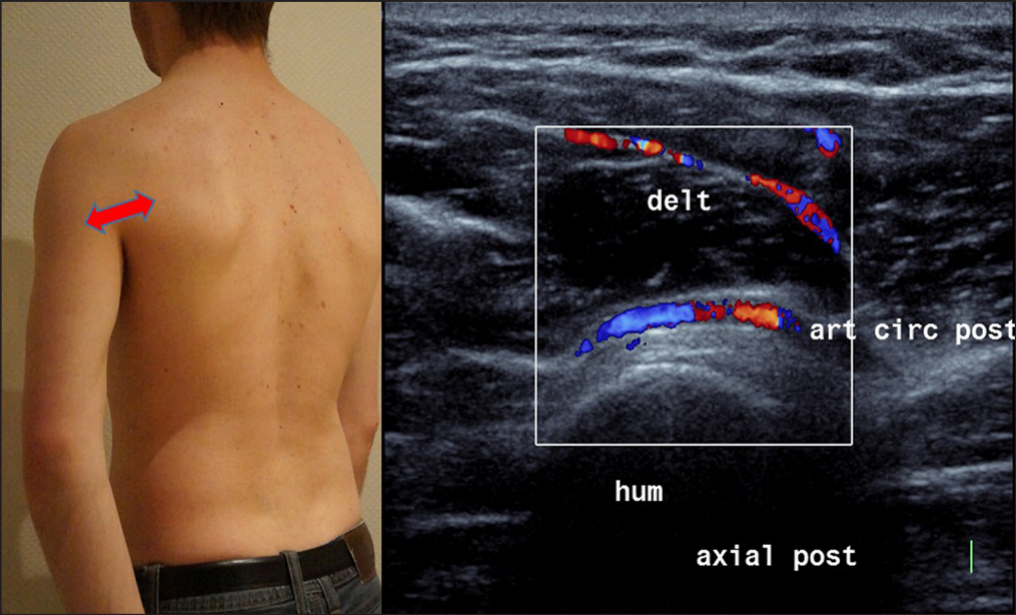
Axial posterior view of the humerus. delt: Deltoid muscle, hum: humerus, art circ post: Axillar artery.

The content analysis:

- A posterior sagittal cross section helps locating the infraspinatus and Teres Minor (in abduction and internal rotation, like with the infra spinatus analysis). The circumflex artery and the posterior axillary nerve are opposite the lower portion of Teres Minor in a small echoic fatty angle.
- Posterior axial section allows identifying the circumflex artery in an axial plane. Color Doppler can be useful to confirm the vascular origin (Figure 7 and Figure 8).

## The Triceps Brachii

**Figure 9:**
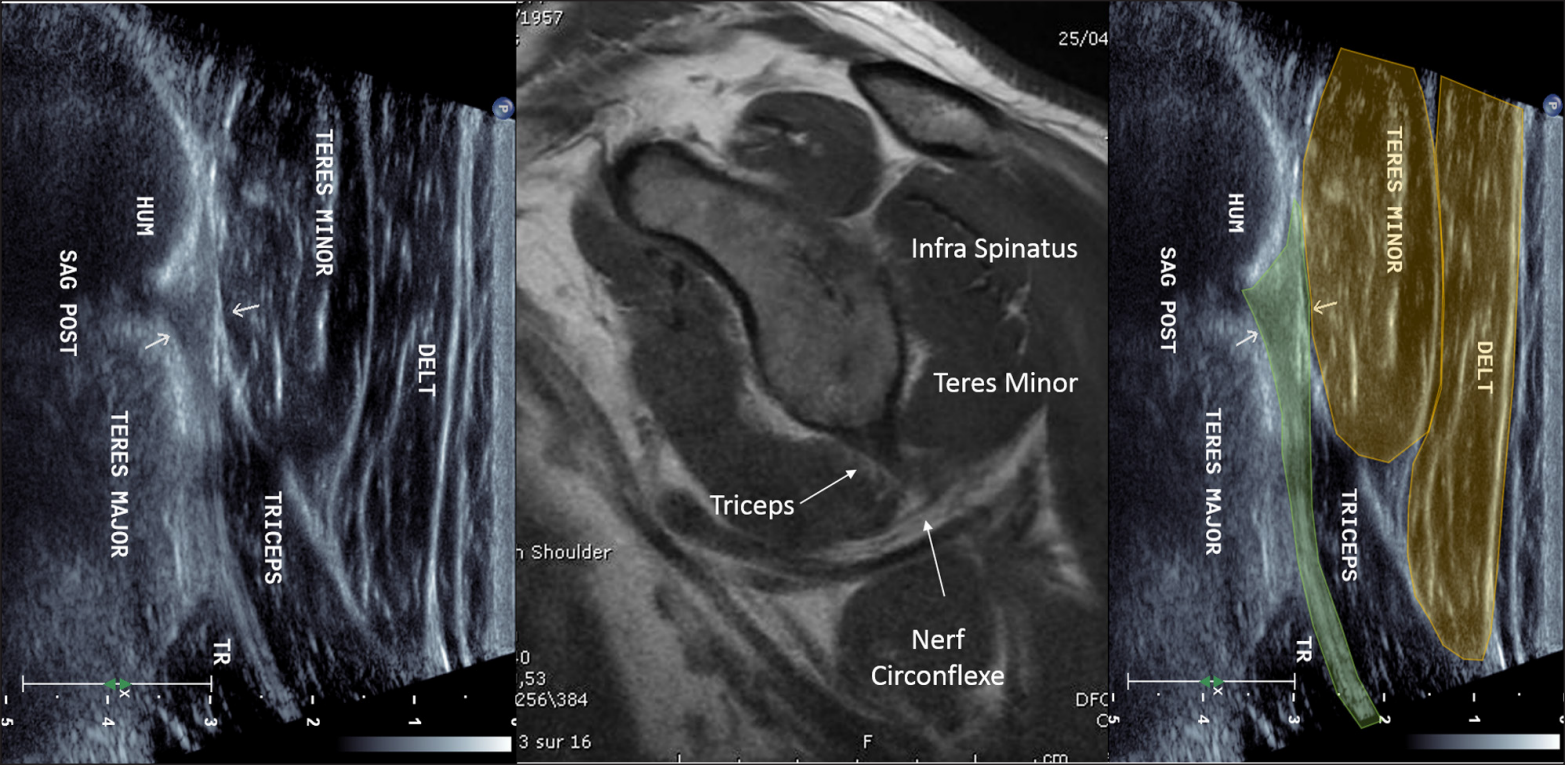
Sagittal view of the triceps tendon with MRI correlation. HUM: Humerus, TR: Triceps muscle, Nerf circonflexe: circumflex nerve.

The long head of the Triceps Brachii originates in the infra-glenoid fossa of a short tendon [2]. The tendon is examined with the arm in abduction and external rotation (similar to the infra spinatus tendon). The analysis is made arms along the body in internal rotation or hand onto the contralateral shoulder. Sagittal cross sections provide lots of informations and the tendon appears to be short, hyper echoic and fibrillar (Figure 9). The probe is in the axis of the humerus searching for the lower side of the glenoid.

**Figure 10:**
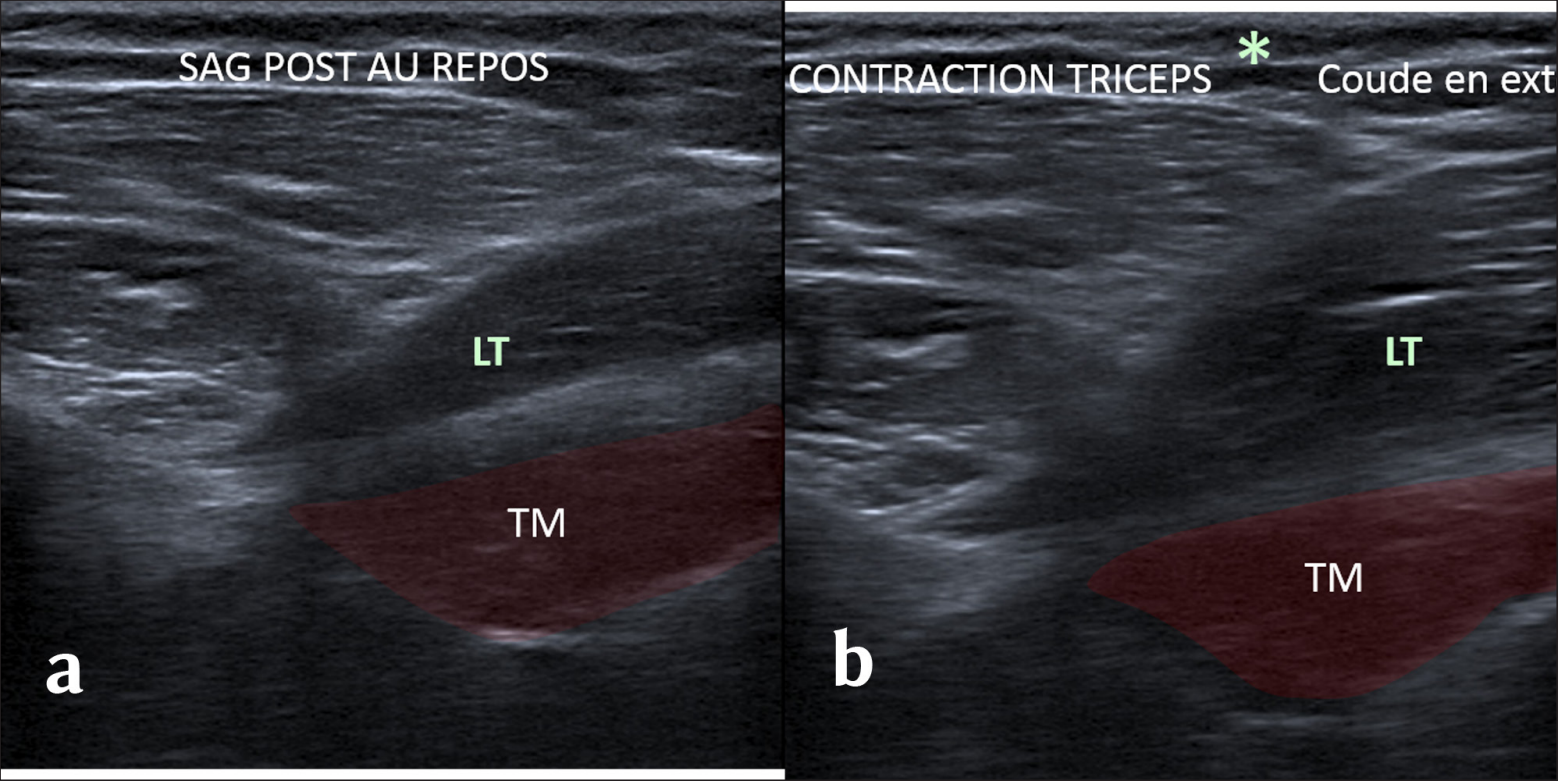
Sagittal view without (a) and with (b) Tricepscontraction with elbow in extension. TM: Teres Major, LT: long head of the triceps.

In axial cross section, the tendon of the Triceps Brachii shows the lateral edge of the Velpeau Quadrilateral Space. Adjunct tendon bundles are not identifiable. The first layer shows the Teres Minor muscle and a small triangle with the fatty axillary nerve and posterior circumflex artery. The dynamic maneuvers with tricipital contraction make the difference between the long head of the Triceps Brachii muscle of the underlying Teres Major (Figure 10).

## The myotendinous junction of the supra spinatus

**Figure 11:**
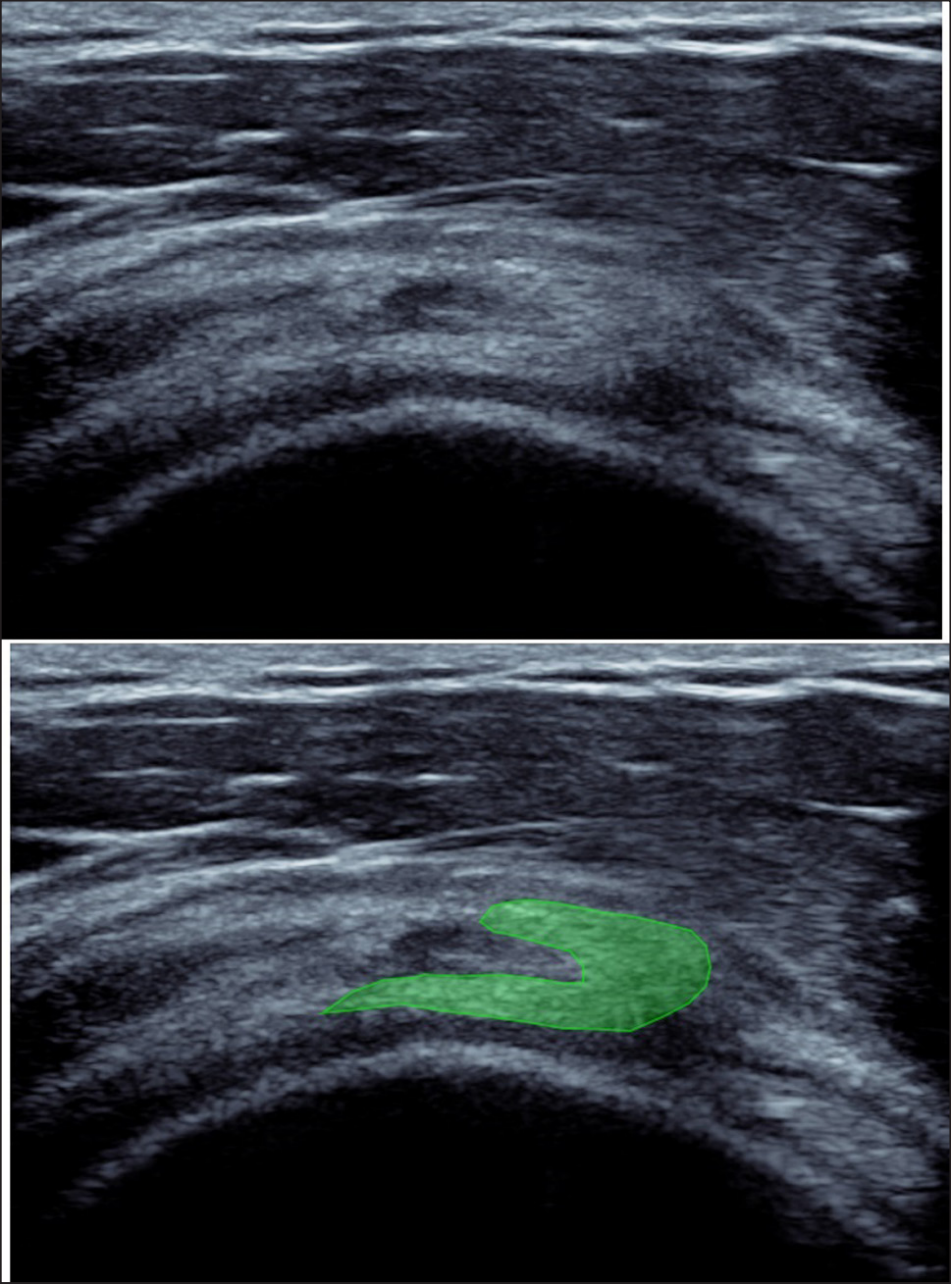
Sagittal view of the rotatorcuff with “coma” appearance of the myotendinous junction of the supra spinatus.

At the myotendinous junction of the supra spinatus (2cm proximal to the insertion), an anterior tendinous complex and a posterior aponeurotic portion. This was analyzed in a conventional position, hand on the buttock with shoulder on retro drive through inside-out sagittal cross sections with a “comma” appearance. This may be the root of symptomatic myotendinous crack, responsible for posterior shoulder pain (Figure 11) [9].

## Round the Coracoid process: short head of the Biceps brachii, Coracobrachialis et Pectoralis minor

The coracoid process (except in traumatic context of fracture in which ultrasound is in our experience a very simple and efficient examination) is the site of insertion on its lateral plane of the short head the biceps brachii and coracobrachialis with very few pathologies type tendinosis or crack. Calcifications with hydroxyapatite resorption can arise yet.

On its medial plane the pectoralis minor inserts medially to the horizontal portion of the coracoid process, with no specific pathology.

**Figure 12:**
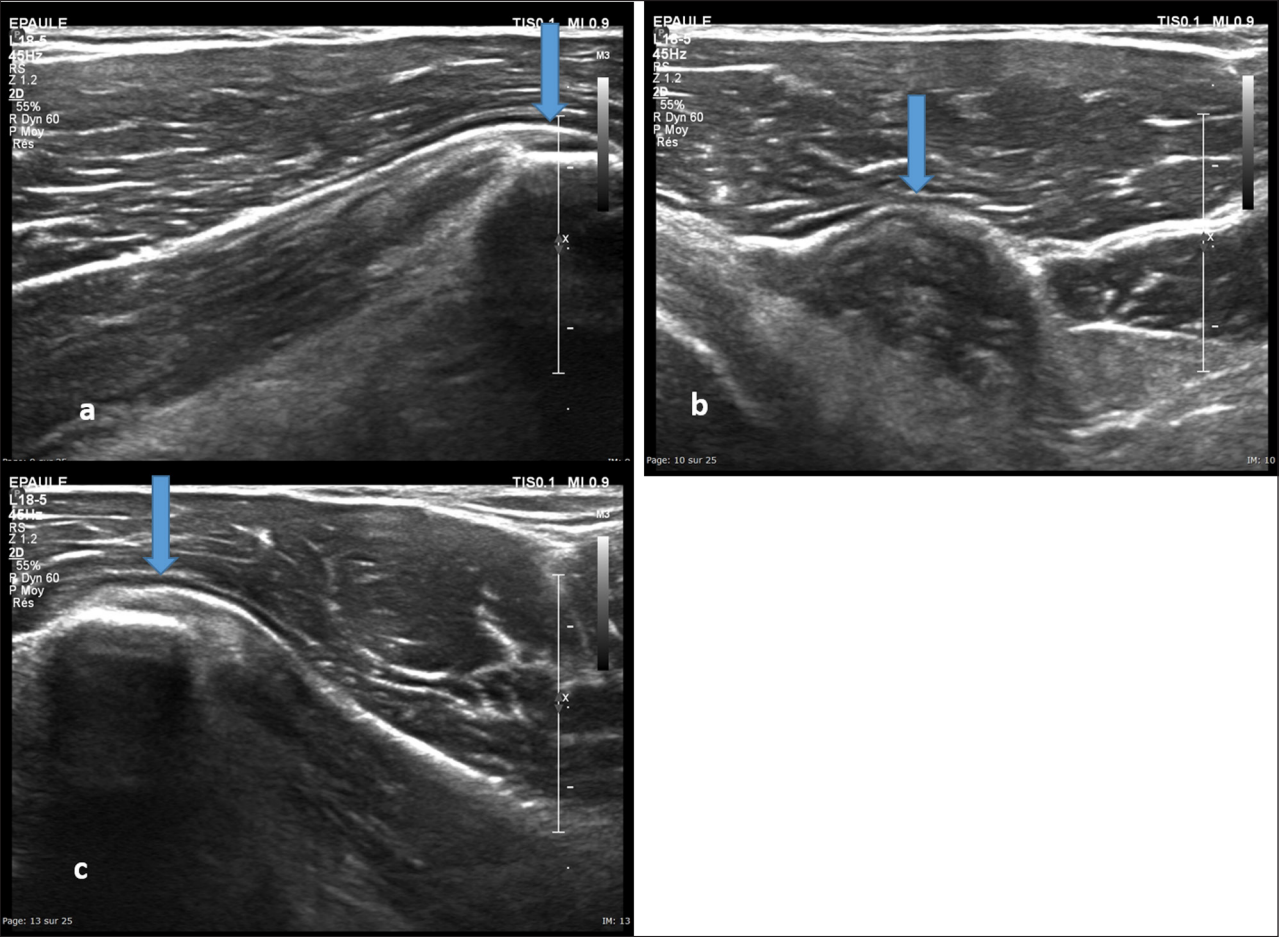
Axial view of the coracoid process with enthesis of the short head of the biceps brachii (a and b) and the coracobrachialis (c).

**Figure 13:**
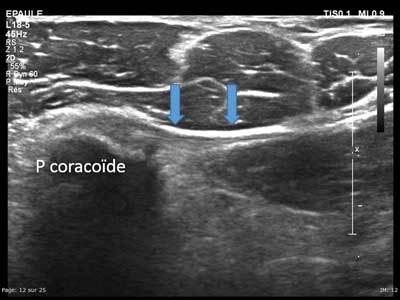
Axial view of the medial part of the coracoid process with enthesis of the pectoralis minor (arrows).

The ultrasound-guided exploration is performed in axial plane, arm in external rotation and then externally down to go on with the Biceps brachii and Coracobrachialis biceps muscles or internally to study the pectoralis minor (Figures 12–13).

## The Musculocutaneous nerve

The Musculocutaneous nerve originates from the lateral cord of the Brachial Plexus. It runs between the two heads of the Coracobrachialis muscle at the upper third of the arm. Then it arises front to the Brachialis muscle and back to the Biceps brachii muscle [10].

Anteriorly to the elbow joint, it inclines laterally to the distal tendon of the Biceps Brachii with a semicircular-shape path. The nerve lies then on the deep slope of the cephalic vein.

At the forearm, it becomes the lateral antebrachial cutaneous nerve and pierce deep to the cephalic vein. Anatomic variations are possible. Its lesion is responsible for hypoesthesia of the lateral plane of the forearm (at its middle third and more proximal to the Wartenberg neuritis).

**Figure 14:**
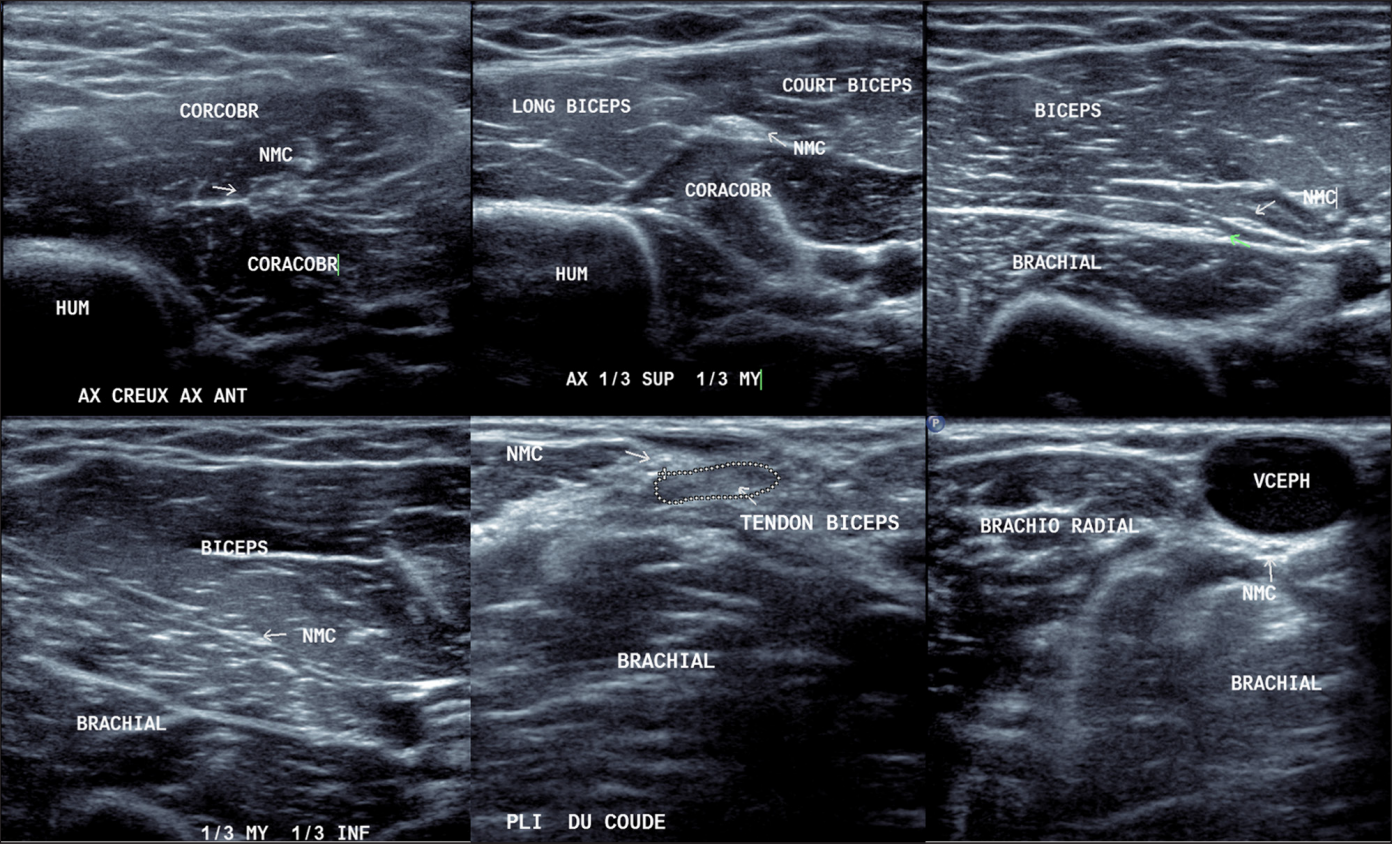
Axial view of the musculocutaneous nerve from the axillar area to the elbow (a to f). NMC: Musculocutaneous nerve, Coracobr: coracobrachial muscle, Hum: Humerus, Long Biceps: Long head of the biceps, Court biceps: short head of the biceps, Brachial: Brachial muscle, Brachio-radial: Brachio-radial muscle, VCEPH: Cephalic vein.

Exploration is done arms along the body with axial cross sections according to the elevator’s technique identifying the Coracobrachialis nerve that crosses back and forward (Figure 14 a–f).

## The Pectoralis Major muscle

**Figure 15:**
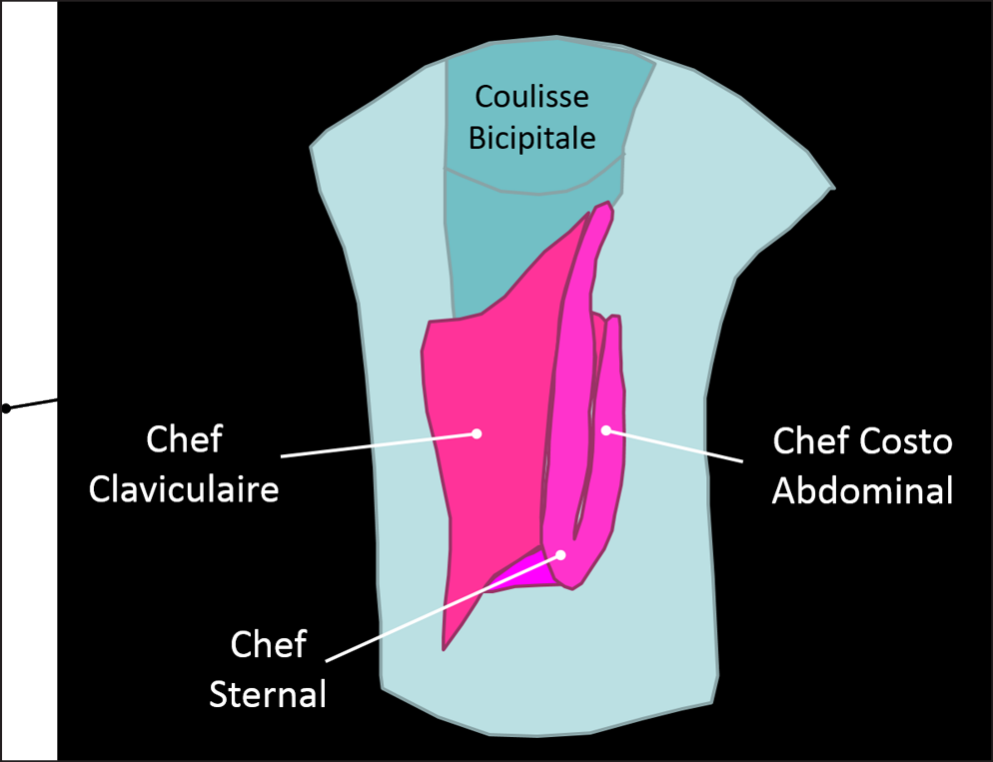
Pectoralis Major tendon on the humerus. Chef Sternal: sternal muscle, Chef Claviculaire: Clavicular muscle, Chef Costo Abdominal: Costo-abdominal muscle, Coulisse Bicipitale: Bicipital area.

As adductor, medial rotator and antepulsion of the arm, the Pectoralis Major consists of three portions converging to the bottom and side of the bicipital groove (clavicular head (2/3 medial clavicle), sternal head (1/2 > sternum), Abdominal costo portion (2–6th ribs’ costal cartilages, the external oblique fascia) [2]. The enthesis has a U-shape with the sternal clavicular heads inserting distally forward while the costo abdominal insertion fits in backwards and upwards. However neither ultrasound nor MRI can demonstrate the multilamellar appearance of the tendon (Figure 15).

**Figure 16:**
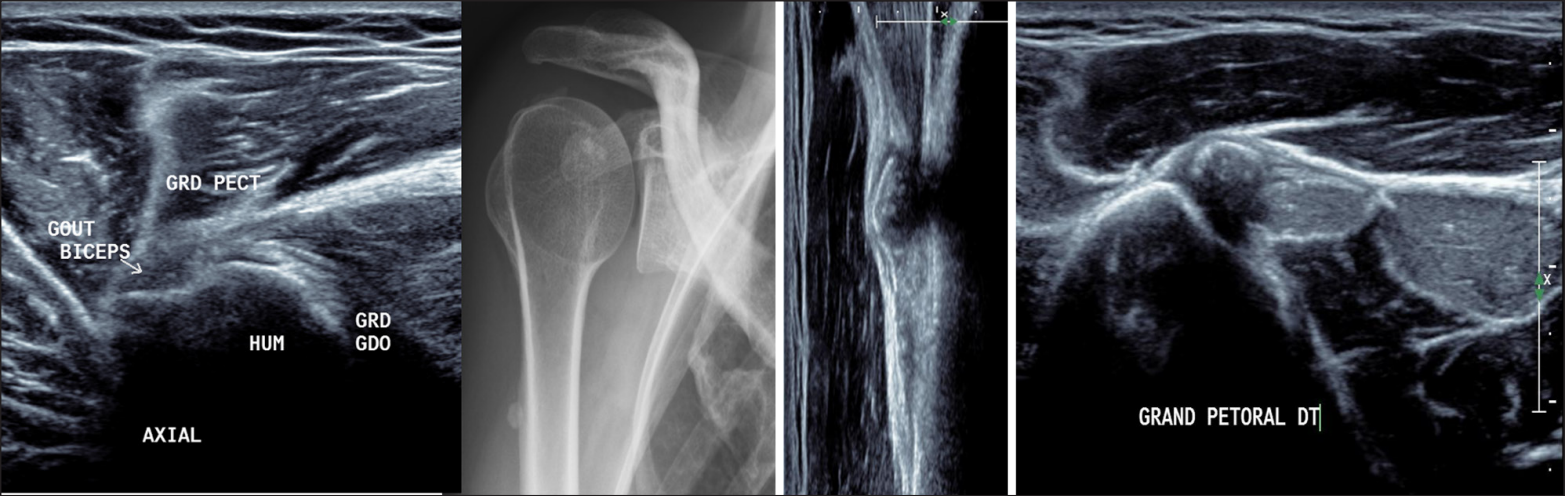
Axial view of the normal anterior part of the humerus in external position (a) with hydroxyapatite deposits in X-rays (b) and US in sagittal and axial view (d). GRD/GDO: Teres Major/Latissimus Dorsi, GRD PECT: Pectoralis Major, HUM: Humerus, Court Biceps: Short head of the biceps.

Injuries are mainly traumatic onto the tendon insertion or myotendinous junction. But in the context of previous shoulder pain, enthesopathies are also described [11]. At ultrasound exploration is performed in sagittal plane from the humeral insertion to the sternum searching for an architectural disorganization, hematoma or asymmetry compared to the opposite side plus by contraction and the ABER position (abduction external rotation) (Figure 16).

## The Deltoid muscle

**Figure 17:**
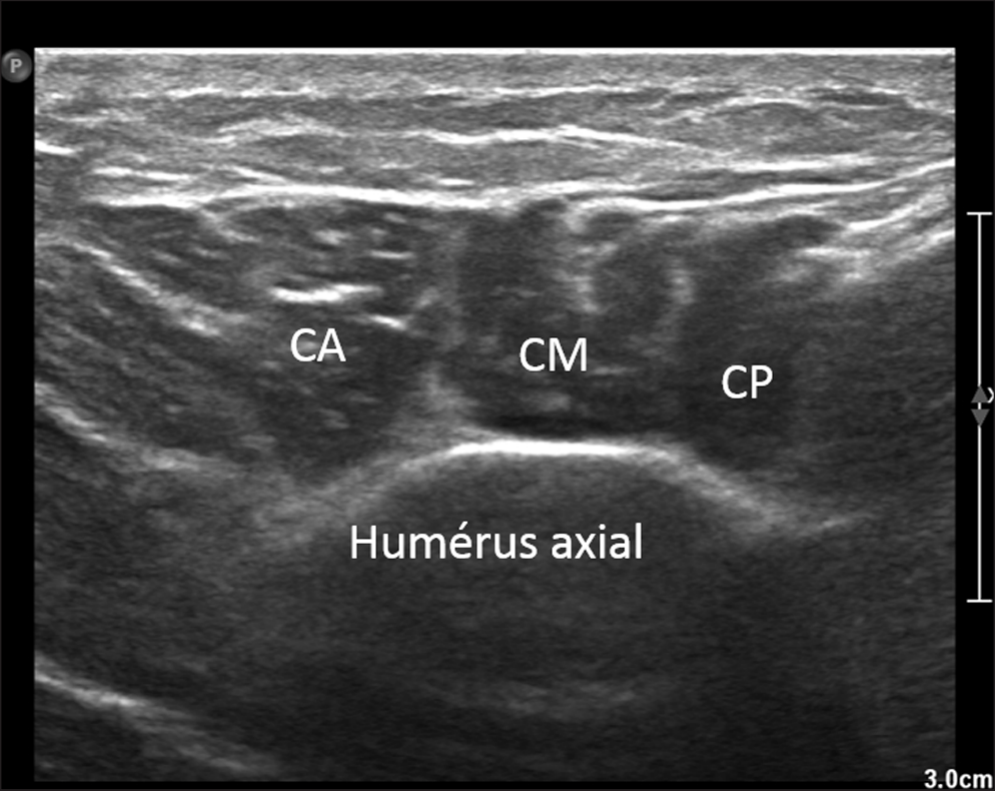
Axial view of the distal and lateral part of the deltoid muscle. CA: Anterior chief, CM: Medium chief, CP: Posterior chief.

It swathes and stabilizes the glenohumeral joint and consists in three branches converging towards the lateral tuberosity of the proximal humerus (Figure 17) [2].

A lesion is observed in 0.3% to 9.2% of rotator cuff tears, usually in its middle portion. After (massive) cuff rupture, two mechanisms may occur: ascension of the humeral head with subacromial impingement possibly leading to avulsion; Lateral subluxation of the humeral head causing an impingement between the greater tubercle and the deep surface of the myotendinous junction [12].

**Figure 18:**
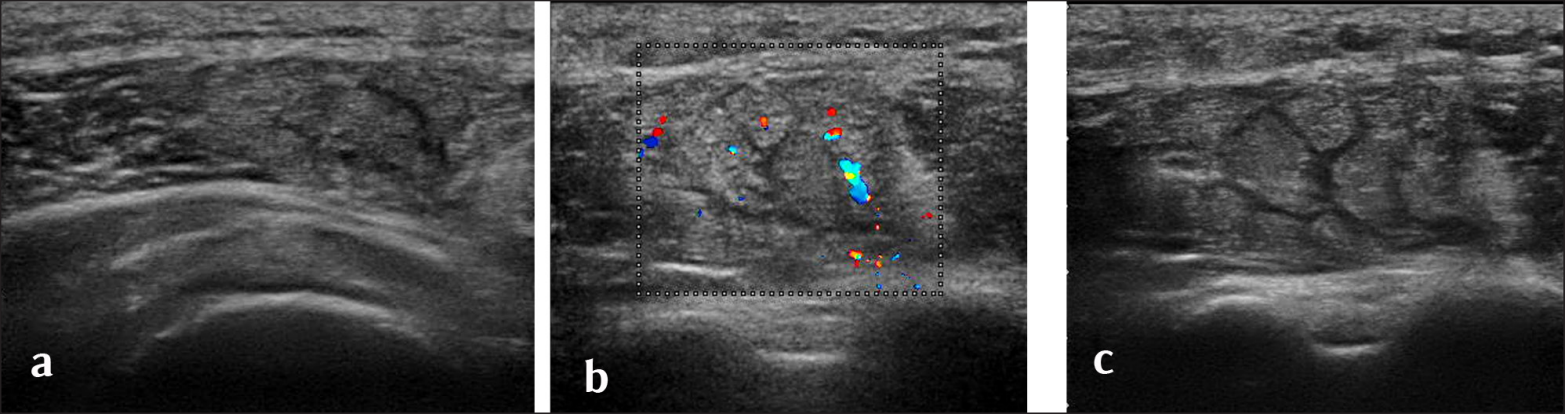
Axial view of a recent traumatic extrinsic lesion of the medium chief of the deltoid with hyperhemia (a–c).

In case of sports injuries, most of the lesions on anterior and middle portions of the Deltoid (motor sports, contact sports, weight training) (Figure 18) [13]. Ultrasound allows an anterior, middle and posterior optimal study of its muscles’ insertions and its acromial and humeral enthesis where apatite deposits may lay.

## Conclusion

This standardized ultrasound-guided examination allows an optimal and reproducible study of resistant shoulder pain and first ultrasound-guided examinations unable to explain the patient’s symptomatology.

## Competing Interests

The authors declare that they have no competing interests.
